# Effect of lighting conditions on implantable collamer lens vault: Influence of anterior chamber and lens parameters

**DOI:** 10.1016/j.heliyon.2024.e37895

**Published:** 2024-09-13

**Authors:** Zhenhao Song, Qi Li, Ying Xiong, Yingyan Mao, Xiaofei Wang

**Affiliations:** a Key Laboratory for Biomechanics and Mechanobiology of Ministry of Education, Beijing Advanced Innovation Center for Biomedical Engineering, School of Biological Science and Medical Engineering, Beihang University, Beijing, China; b Beijing Institute of Ophthalmology, Beijing Tongren Eye Center, Beijing Tongren Hospital, Capital Medical University, Beijing Ophthalmology & Visual Sciences Key Laboratory, Beijing, 100730, China; c Beijing Tongren Eye Center, Beijing Tongren Hospital, Capital Medical University, Beijing Ophthalmology & Visual Sciences Key Laboratory, Beijing, 100730, China; d Beijing Advanced Innovation Center for Big Data-Based Precision Medicine, Beihang University & Capital Medical University, Beijing Tongren Hospital, Beijing, 100730, China; e Eye Hospital, School of Ophthalmology and Optometry and School of Biomedical Engineering, Wenzhou Medical University, Wenzhou, China

**Keywords:** Implantable collamer Lens, Lens vault, Luminance, High myopia, Anterior segment OCT

## Abstract

**Purpose:**

To investigate the change in the vault of the implantable collamer lens (ICL) under dark-to-light conditions and its association with anterior chamber and lens parameters in patients undergoing ICL surgery.

**Methods:**

In 76 eyes from 40 patients, preoperative anterior chamber volume (ACV), pupil diameter (PD), anterior chamber angle, central corneal thickness (CCT), white-to-white (WTW), lens thickness (LT), axial length (AL), spherical equivalent (SE) and patient's age were collected. Postoperative vault, PD and LT were measured under dark and light conditions using anterior segment optical coherence tomography (CASIA2; TOMEY, Japan), and changes and lens displacement under dark-to-light conditions were calculated. Mixed-effects models were used to analyze the correlation between the vault change and the anterior chamber and lens parameters of all subjects and the high-vault subgroup.

**Results:**

The vault under light condition (648.36 ± 304.47 μm) was significantly smaller compared to the vault under dark condition (708.89 ± 316.15 μm). In all patients, vault change increased with the increase of age, lens displacement and PD change; and increased with the decrease of ACV, LT change and baseline vault (under dark condition). In the high-vault subgroup, vault change increased with the increase of CCT, lens displacement and PD change; and increased with the decrease of ACV.

**Conclusions:**

ICL vault changes significantly from dark to light, influenced by age, ACV, PD change, LT change, lens displacement, and baseline vault. A higher baseline vault is correlated with a larger LT change, affecting the levels of accommodation under dark-to-light transition.

## 1 Introduction

Implantable collamer lens (ICL) surgery is a procedure used to correct high myopia of up to −18.00 diopters and astigmatism of up to −6.00 diopters [1]. It involves the placement of an ICL in the ciliary sulcus between the natural crystalline lens and the iris [2]. The vault of the ICL, defined as the distance between the posterior surface of the ICL and the anterior surface of the crystalline lens, is a key parameter in evaluating the procedure's safety [3]. A safe vault value has been suggested to be between 250 and 750 μm [4,5]. An insufficient vault may induce mechanical contact between the ICL and the crystalline lens or inadequate aqueous circulation, potentially causing anterior capsule opacification and cataract. Conversely, an excessive vault may result in a strong mechanical contact between the ICL and iris, causing inflammation and increased intraocular pressure, leading to angle-closure glaucoma and pigment dispersion syndrome [6].

The vault is not a static value and can change over time[[7], [8], [9]] and in response to changes in accommodation status [10]. Typically, the vault decreases gradually after implantation and changes dynamically during accommodation[[10], [11], [12]]. Previous studies have demonstrated that the ICL vault decreases significantly under light conditions. These dynamic changes in response to the constant variations in lighting conditions during daily activities can impact the safety of the ICL. While previous studies have examined the changes in ICL vault under different lighting conditions, they have largely been limited to exploring the relationship between vault and pupil size without considering other structures in the anterior chamber.

With the advancement of optical coherence tomography (OCT) technology, it is now possible to visualize the entire anterior chamber and the full thickness of the crystalline lens, allowing for a more comprehensive examination of factors influencing the ICL vault under different lighting conditions. Therefore, the aim of this study was to investigate the influence of lighting conditions on the vault of the ICL and its influencing factors using anterior segment OCT (AS-OCT).

## 2 Methods

### 2.1 Subject recruitment and clinical examinations

This study included 76 eyes from 40 patients who underwent ICL V4c (STAAR Surgical Company, Monrovia, CA) implantation at Beijing Tongren Eye Center. The four excluded eyes were from subjects for whom OCT data were collected for only one eye. To investigate the characteristics of the high-vault group, half of the 40 patients (20) were purposely selected based on a vault higher than 750 μm. Written informed consent was obtained from all participants. The study had the approval of the Institutional Review Board of Beijing Tongren Hospital and adhered to the tenets of the Declaration of Helsinki.

Inclusion criteria were as follows: age over 18 years, myopia ≥ −0.5D, stable for at least 1 year (defined as progression in refraction of no more than −0.5D per year), corrected distance visual acuity (CDVA) ≥ 20/40, anterior chamber depth (ACD) > 2.8 mm and central corneal endothelial cell count ≥2000 cells/mm^2^. Exclusion criteria were: history of eye surgeries, trauma, other ophthalmic diseases, systemic diseases, or other reasons that could affect following measurement.

Before ICL implantation, anterior chamber parameters, including anterior chamber volume (ACV), pupil diameter (PD), and anterior chamber angle, were measured using Pentacam (Oculus; Wetzlar, Germany). In addition, central corneal thickness (CCT), white-to-white (WTW), lens thickness (LT), and axial length (AL) were measured using Lenstar (LS-900; Haag-Streit AG, Switzerland). Pre- and postoperative spherical equivalent (SE) was measured using an autorefractor (RK-3000; Topcon, Japan).

### 2.2 Optical coherence tomography imaging and analysis

The size of ICL was determined by the ICL formula of STAAR. One week after ICL implantation, the anterior segment of the eye was imaged using AS-OCT (CASIA2; TOMEY, Nagoya, Japan) in the “Lens Movie” mode. By this time, the patient's vault had stabilized [13]. Live OCT imaging was started in a dark environment (light intensity, 0.1 lux). After 1–2 s, a shinning penlight was placed before the fellow eye (light intensity, 5962.8 lux) to capture anterior chamber structure changes under dark-to-light conditions. The light intensity was measured using a spectral irradiance sensor (SIS-20; China). The change in ICL vault from dark to light conditions in a typical eye is shown in the supplemental video.

**Fig. 1:**
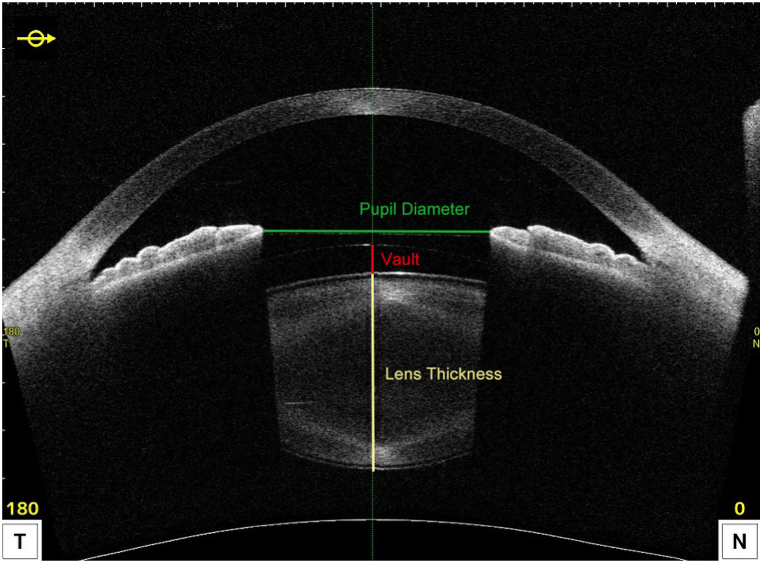
Dewarped OCT image of the anterior segment showing measurements of vault, pupil diameter and lens thickness.

The obtained OCT images were first dewarped to correct image distortion inherited from the imaging system. Measurements of vault, PD, and LT were taken under two lighting conditions. Dewarping and measurements of parameters were performed using the CASIA2 software. Measurements of patient's vault, LT and PD was shown in Fig. 1.

The baseline vault was defined as the ICL vault under dark conditions. Vault change, or Δvault, was defined as the vault under light conditions minus the baseline vault. A negative Δvault value indicates a reduce in vault under dark-to-light conditions and vice versa. Similarly, PD change and LT change were calculated as the difference between under light conditions and their respective baseline values in the dark conditions.

Anterior surface displacement of the lens was measured as the relative movement of its anterior surface with respect to the posterior surface of the cornea under dark-to-light conditions, with positive values denoting posterior displacement and negative values indicating an anterior shift. Lens displacements were then calculated as half of LT change plus lens anterior surface displacements, representing a shift in the lens position respect to the cornea and mitigating the effects of LT change. Positive values denote a posterior shift, while negative values indicate an anterior shift.

### 2.3 Statistical analysis

The statistical analysis was initially performed on all subjects. Subsequently, a separate analysis was performed for patients with a vault measurement greater than 750 μm. The normality of the vault was assessed using the Kolmogorov-Smirnov (KS) test. For parameters with normal distributions, a paired *t*-test was used to determine whether there was a significant difference between the two data groups.

We used mixed-effects models to account for inter-eye correlation [14]. Both univariate and multivariate mixed-effects models were constructed to examine the potential correlation between each ocular parameter and Δvault. Parameters with p < 0.2 in the univariate mixed-effects model were included in the multivariate mixed-effects model.

All statistical analyses were conducted using the "Statsmodels" library and “Scipy” library in Python (version 3.10.4, python.org). A p-value of less than 0.05 was considered statistically significant.

## 3 Results

### 3.1 Demographic and clinical characteristics

This study included a total of 76 eyes from 40 subjects who had undergone ICL surgery. Of these, 33 eyes from 20 patients exhibited high vault and were grouped into a subset for analysis. The demographic and ocular parameters of all subjects and patients in high vault group are shown in Table 1.

**Table 1:** Demographic and ocular characteristics of all subjects and those of the high-vault subgroup.

Parameter	Mean (all subjects)	Range (all subjects)	Mean (high-vault group)	Range (high-vault group)
Baseline vault (μm)	707.89 ± 316.15	[81.00, 1550.00]	993.21 ± 200.11	[758.00, 1550.00]
Vault (light) (μm)	648.36 ± 304.47	[20.00, 1588.00]	919.88 ± 214.60	[625.00, 1588.00]
Vault change (Δvault) (μm)	−59.54 ± 50.96	[-187.00, 56.00]	−73.33 ± 53.05	[-187.00, 38.00]
ICL Size	129.76 ± 3.98	[121, 137]	131.73 ± 2.60	[126, 137]
Age (year)	30.71 ± 5.81	[21, 44]	29.06 ± 3.98	[21, 35]
Preoperative CCT (μm)	516.20 ± 34.45	[450.0, 584.0]	523.67 ± 39.27	[450.0, 584.0]
Preoperative Anterior chamber angle	39.50 ± 4.74	[29.9, 50.9]	39.66 ± 4.26	[30.4, 50.9]
Preoperative AL (mm)	26.74 ± 1.21	[24.29, 31.02]	26.94 ± 1.00	[25.35, 29.66]
Preoperative ACV (mm³)	206.68 ± 27.16	[124, 262]	214.73 ± 19.87	[184, 251]
Preoperative WTW (mm)	11.90 ± 0.38	[11.12, 12.79]	12.06 ± 0.33	[11.53, 12.79]
Preoperative LT (mm)	3.70 ± 0.27	[3.22, 4.44]	3.58 ± 0.23	[3.22, 4.05]
LT change (μm)	−2.82 ± 33.17	[-86, 78]	−6.91 ± 30.97	[-74, 68]
Lens displacement (μm)	5.37 ± 18.96	[-56.5, 52.5]	3.03 ± 21.16	[-56.6, 52.5]
Preoperative PD (mm)	3.16 ± 0.49	[2.07, 4.38]	3.26 ± 0.37	[2.60, 3.99]
PD change (mm)	−1.21 ± 0.41	[-2.06, −0.07]	−1.25 ± 0.47	[-1.92, −0.07]

ICL = implantable collamer lens; CCT = central corneal thickness; AL = axial length; ACV = anterior chamber volume; WTW = white-to-white; LT = lens thickness; PD = pupil diameter.

Baseline vault and vault (light) are normally distributed (KS test p > 0.05). Baseline vault and vault (light) are significantly different (Paired *t*-test p < 0.05). Parameters measured before the operation were explicitly indicated in the table. If not specified, parameters were measured postoperatively.

### 3.2 The relationship between vault change and its determinants

**Fig. 2:**
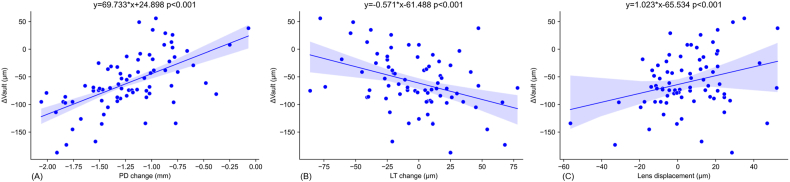
Scatter plots demonstrating some correlations between Δvault and ocular parameters for all subjects. A negative Δvault value indicates a reduce in ICL vault and vice versa. LT: lens thickness; PD: pupil diameter. The regression equation is from univariate linear mixed models, but these correlations still exist in the multivariate analysis. (A) Association between Δvault and PD change; (B) association between Δvault and LT change; (C) association between Δvault and lens displacement.

Univariate correlation analysis demonstrated that Δvault was significantly correlated with age, ACV, PD change, LT change, lens displacement and baseline vault (Table 2 and Fig. 2). Multivariate correlation analysis indicated that Δvault was independently correlated with age, PD change, lens displacement and LT change (Table 2).

**Table 2:** Linear mixed models: Δvault with demographic and ocular parameters.

	Univariate				Multivariate			
	β	p-value	β (high-vault group)	p-value (high-vault group)	β	p-value	β (high-vault group)	p-value (high-vault group)
Age	3.360	**0.002**	1.374	0.639	2.444	**0.019**		
Size of ICL	−2.172	0.204	0.373	0.929				
CCT	0.324	0.098	0.539	**0.041**	0.066	0.672	0.090	0.610
Anterior chamber angle	−0.822	0.564	−0.415	0.856				
AL	−3.719	0.496	−9.952	0.343				
ACV	−0.714	**0.002**	−1.221	**0.010**	0.059	0.778	−0.767	**0.024**
LT	42.618	0.097	21.854	0.646	−23.268	0.325		
WTW	3.804	0.837	54.490	0.059			32.377	0.100
PD	−14.989	0.250	−5.404	0.837				
PD change	69.733	**0.000**	76.045	**0.000**	64.115	**0.000**	53.011	**0.000**
LT change	−0.571	**0.000**	−0.228	0.339	−0.444	**0.000**		
Lens displacement	1.023	**0.000**	0.882	**0.020**	0.875	**0.000**	0.383	**0.002**
Baseline vault	−0.053	**0.008**	0.025	0.612	−0.035	0.057		

Bold values indicate p < 0.05. ICL = implantable collamer lens; CCT = central corneal thickness; AL = axial length; ACV = anterior chamber volume; WTW = white-to-white; LT = lens thickness; PD = pupil diameter.

In the multivariate analysis, a 1-year increase in age was associated with a 2.444 μm increase in Δvault, a 1 mm increase in PD change was associated with a 64.115 μm increase in Δvault, and a 1 μm increase in lens displacement was associated with a 0.875 μm increase in Δvault. Conversely, a 10 mm³ increase in ACV and a 1 μm increase in LT change were associated with a decrease in Δvault by 0.59 μm, and 0.444 μm, respectively. Additionally, a 100 μm increase in baseline vault and resulted in a 3.5 μm decrease in Δvault.

### 3.3 The relationship between vault change and its determinants in the high-vault group

In the high vault subgroup, univariate correlation analysis demonstrated that Δvault was significantly correlated with CCT, ACV, lens displacement and PD change (Table 2). Multivariate correlation analysis indicated that Δvault was independently correlated with ACV, lens displacement and PD change (Table 2). In the multivariate analysis, a 10 μm increase in CCT was associated with a 0.90 μm increase in Δvault, a 1 mm increase in PD change was associated with a 53.011 μm increase in Δvault, and a 1 μm increase in lens displacement was associated with a 0.383 μm increase in Δvault. Conversely, a 10 mm³ increase in ACV was associated with a 7.67 μm decrease in Δvault.

### 3.4 Lens displacements and changes in lens thickness

The mean lens displacement was 5.37 ± 18.96 μm, with a range of −56.5 μm–52.5 μm. Mean LT change was −2.82 ± 33.17 μm, with a range of −86 μm–78 μm. Univariate analysis showed a significant correlation between lens displacement and LT change.

**Fig. 3:**
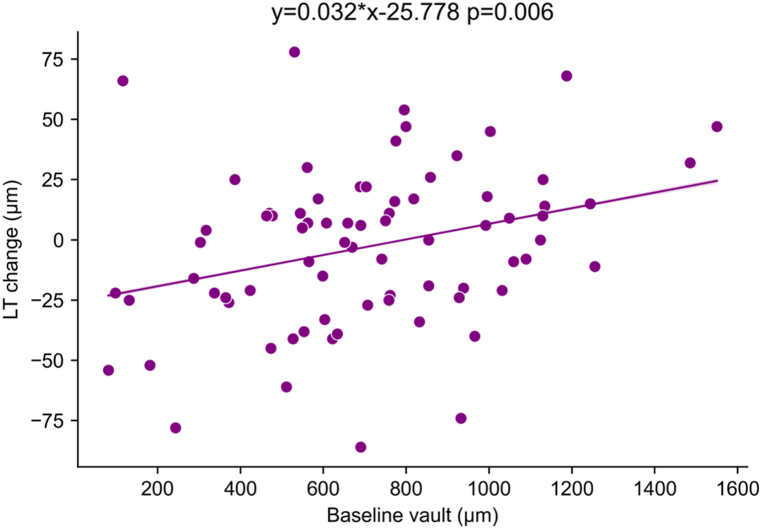
Scatter plots demonstrating correlations between LT change and baseline vault for all subjects. A negative LT change value indicates the thinning in LT change and vice versa. LT: lens thickness. The regression equation is from univariate linear mixed models, but this correlation still exists in the multivariate analysis.

Corrected for age, sex, AL and preoperative LT, LT change also has a significant correlation with baseline vault (p = 0.026) (See supplemental Table A). The scatter plot with linear regression model is show in Fig. 3.

## 4 Discussion

In this study, we evaluated the dynamic changes in ICL vault under dark-to-light conditions and their correlation with the structural characteristics of the eye. We found that ICL vault changed significantly under dark-to-light conditions and was influenced by age, ACV, PD change, LT change, lens displacement and baseline vault. A higher baseline vault is correlated with a larger LT change, and thus, affects the accommodation level under dark-to-light conditions.

We were able to measure LT, lens displacements and other anterior segment parameters simultaneously using the new anterior segment OCT, which has not been studied before. Our results showed that both thickening and anterior displacement of the lens are significant factors influencing vault change under dark-to-light conditions (Table 2). This is straightforward as increase of both factors could bring the ICL closer to the lens. We further examined the relative importance of these two factors on Δvault through linear mixed model analysis (See supplementary Table B). We found that lens displacement exerts a greater influence than LT change. While previous studies focused on the changes of pupil diameter on Δvault, our findings also highlight lens changes as significant factors that needs to be considered.

We also observed a significant difference in LT change between high-vault patients and those with a lower vault. A significant correlation between LT change and baseline vault (Fig. 3) was found across all subjects, even after adjusting for age, sex, axial length and preoperative LT. Specifically, lenses in patients with a high baseline vault exhibited greater thickening under dark-to-light conditions. The exact reason for this correlation remains unclear, but we speculate that it stems from the impact of ICL implantation on lens thickness change and accommodation. In subjects with a high vault, the iris is pushed upwards, necessitating iris movement along a slope when the pupil diameter decreases in dark-to-light transition. As a result, the iris tip moving along the slope faces more resistance, requiring a stronger contraction of the sphincter muscle than a flat iris would need to achieve the same pupil diameter. This larger contraction force requires greater nerve stimulation. Given that both the pupillary sphincter and the contraction of the ciliary muscles are controlled by the parasympathetic nerve [15], this implies a greater contraction force of the ciliary muscle as well, leading to lens thickening. As accommodation reflex includes coordinated changes in vergence, lens thickness, and pupil size, it is possible that alterations in the mechanical environment of the iris due to ICL implant could potentially affect accommodation. The disruption of accommodation function could affect accommodative accuracy, possibly causing visual discomfort or fatigue. Future studies are needed to systematically explore the effects of ICL implantation on the eye's accommodative function to improve ICL procedure outcomes.

Previous studies have reported a significant decrease in both vault and PD under light conditions, and that vault is significantly correlated with PD during this process [13,16]. Our results support these findings, as we observed a significant correlation between Δvault and PD change under dark-to-light conditions in univariate and multivariate linear mixed models for all patients and the high-vault group. This suggests that the mechanical interactions between the iris and the ICL during pupillary constriction under light conditions may be the primary cause of the change in ICL position. A larger change in PD may correlate with a greater contraction force of the pupillary sphincter and lead to a stronger compression of the ICL, resulting in a larger backward movement of the ICL.

In the univariate analysis, we found that Δvault was significantly correlated with the baseline vault. A higher baseline vault tends to result in a greater relative posterior movement of the ICL. This may also be attributed to the potential mechanical interactions between the iris and ICL. A higher vault may introduce a pre-stress on the iris as it pushes the iris upwards under normal conditions. During pupil constriction, this pre-stress might in turn result in a larger contact force between the iris and ICL, leading to a greater backward movement of the ICL. This may also explain why a high baseline vault tends to decrease more over time [17,18].

Age appears to be associated with a smaller relative posterior movement of the ICL. This might be due to aging causing a reduction in pupil size under dark condition [19], a weaking of lens transmission [20] and a decline in peripheral somatic nerve function [21], leading to decreased light intake and altered pupil light-reflex under light condition [22]. Additionally, older patients typically have a lower baseline vault (See supplementary Figure A), resulting in less mechanical interaction between the iris and the ICL as discussed above, leading to a smaller posterior movement of the ICL.

In the high-vault group, a larger ACV is independently correlated with a larger posterior displacement of the ICL. While the exact reason for this correlation is unclear, we speculate that it might be related to the pre-stress of the iris after surgery, similar to the effects of the baseline vault. Specifically, previous studies have shown that postoperative ACV will decrease significantly, due to the forward movement of the iris under the push from the ICL [23]. Furthermore, a larger preoperative ACV leads to a larger change in ACV, which means ICL provides a greater push on the iris, resulting in a larger baseline vault as evidenced by the relationship between the baseline vault and preoperative ACV (See supplementary Figure B). The more pre-stressed iris may exert more pressure on the ICL during pupil constriction, leading to a larger backward movement of the ICL, as discussed above. Additionally, ACV is correlated with several other factors, including iris volume [24]. A thicker iris may cause an increased passive resistance from the iris stroma during constriction, or reduced iris mobility due to increased friction forces within the anterior chamber, leading to a decreased pupillary response. These factors may contribute to the differences in Δvault in subjects with varying ACV [25]. However, these speculations need further studies to be confirmed.

There was no significant correlation between Δvault and AL. Despite previous studies have shown that AL was negatively correlated with LT and positively correlated with ACD [26], it appears that AL does not have a significant impact on Δvault. This may be because the increase in AL is mainly attributed to growth of the vitreous chamber [27]. The impact of AL increase on the anterior chamber structure may not be substantial enough to affect Δvault during dark-to-light conditions.

There are several limitations in this study that warrant further discussion. First, the size of the sample is relatively small and only uses a single light condition, lacking comparisons. Second, some ocular parameters were not measured dynamically, and there may be a lack of evidence on how to explain the change in vault under dark-to-light conditions.

## 5 Conclusions

This study provides important insights on the factors influencing Δvault under dark-to-light conditions and highlights the necessity of considering dynamic vault changes when evaluating ICL implantation safety. Our findings indicate that Δvault is significantly associated with age, baseline vault, ACV, PD change, LT change and lens displacement. A higher baseline vault is correlated with a more substantial LT change, thereby affecting the level of accommodation under dark-to-light conditions. Understanding these dynamics is crucial for optimizing ICL procedures, particularly given the constant variations in lighting in daily life.

In clinical practice, it is essential to incorporate the identified ocular parameters into preoperative assessments to enhance surgical outcomes and patient management, especially in the context of dynamic vault changes. Further research is warranted to refine ICL implantation techniques that address these complex interactions. By incorporating these considerations, clinicians can better anticipate and manage the effects of ICL implantation, ultimately leading to more successful and personalized patient care.

### Ethics and content declarations

This study was reviewed and approved by the Institutional Review Board of Beijing Tongren Hospital, with the approval number: TRECKY2020-030, dated March 1, 2020.

All patients (or their proxies/legal guardians) provided informed consent to participate in the study. All patients (or their proxies/legal guardians) provided informed consent for the publication of their anonymized case details and images.

## Data availability statement

The data that support the findings of this study are available from the corresponding author, upon reasonable request.

## Sources of funding

This research was supported by the National Natural Science Foundation of China (12272030, 12472304), Beijing Natural Science Foundation (Z240017), National Key R&D Program of China (2023YFC2410404) and the Fundamental Research Funds for the Central Universities.

## CRediT authorship contribution statement

**Zhenhao Song:** Writing – original draft, Visualization, Formal analysis, Data curation. **Qi Li:** Visualization, Formal analysis, Data curation. **Ying Xiong:** Writing – review & editing, Resources, Data curation, Conceptualization. **Yingyan Mao:** Writing – review & editing, Resources, Investigation, Data curation, Conceptualization. **Xiaofei Wang:** Writing – review & editing, Supervision, Project administration, Methodology, Conceptualization.

## Declaration of competing interest

The authors declare that they have no known competing financial interests or personal relationships that could have appeared to influence the work reported in this paper.
