# Supplementary material for: Effect of lighting conditions on implantable collamer lens vault: Influence of anterior chamber and lens parameters
Source: Heliyon. 2024 Sep 13;10(18):e37895. doi: 10.1016/j.heliyon.2024.e37895 (PMC11420470; doi:10.1016/j.heliyon.2024.e37895)
Supplement: Multimedia component 1 [file mmc1.docx]

**Supplementary Materials**

**Table A.** Linear mixed models: LT change with baseline vault, age, sex, AL and preoperative LT.

|  | β | p-value |
| --- | --- | --- |
| Baseline vaults | 0.034 | **0.026** |
| Age | 0.265 | 0.727 |
| Sex | 10.001 | 0.280 |
| AL | 3.507 | 0.268 |
| LT | -6.435 | 0.740 |

P<0.05 represents significant effect (in bold).

**Table B.** Linear mixed models: Δvault with LT change and lens displacement.

|  | β | p-value |
| --- | --- | --- |
| LT change | -0.445 | **0.002** |
| Lens displacement | 0.815 | **0.002** |

P<0.05 represents significant effect (in bold).


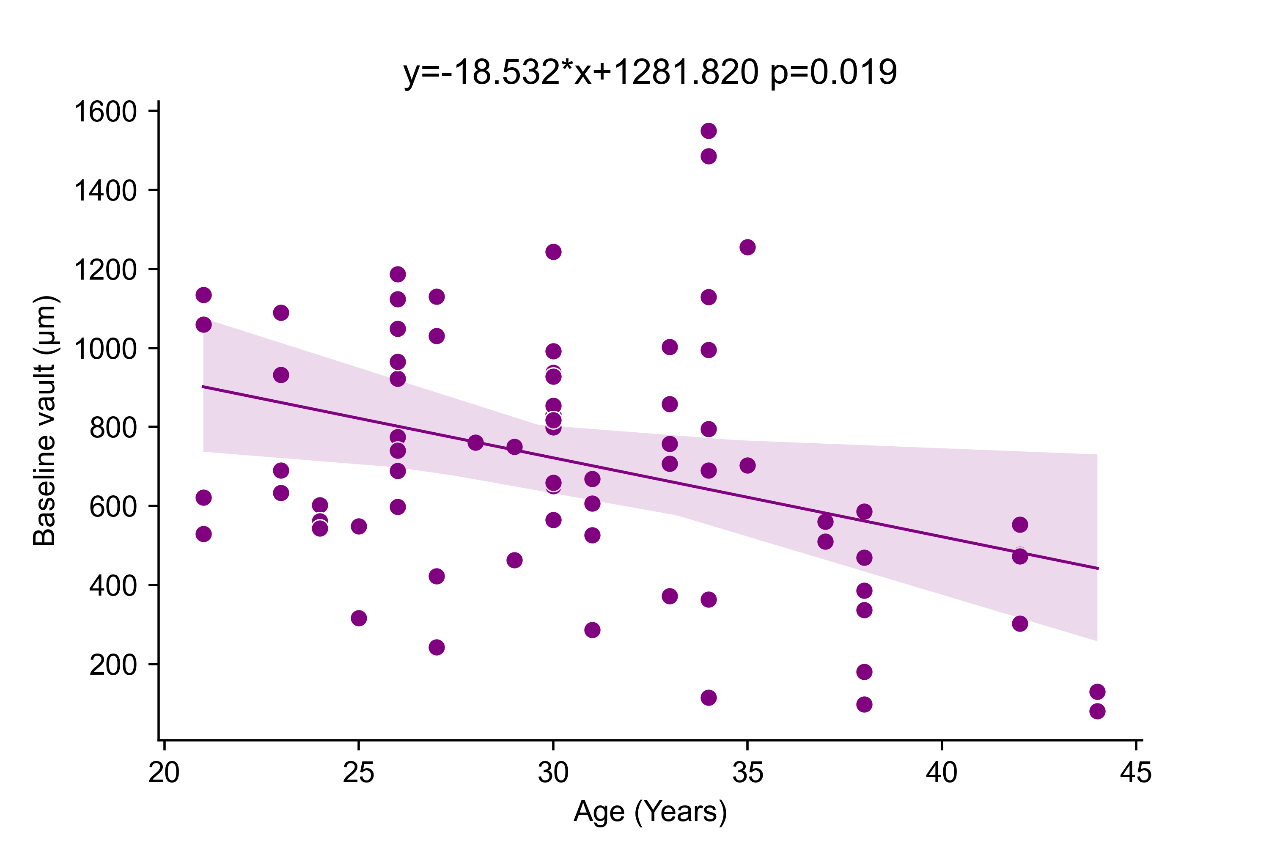


**Figure A.** Scatter plots demonstrating correlations between baseline vault and age. A 1-year increase in age was associated with a 18.53 μm decrease in baseline vault.


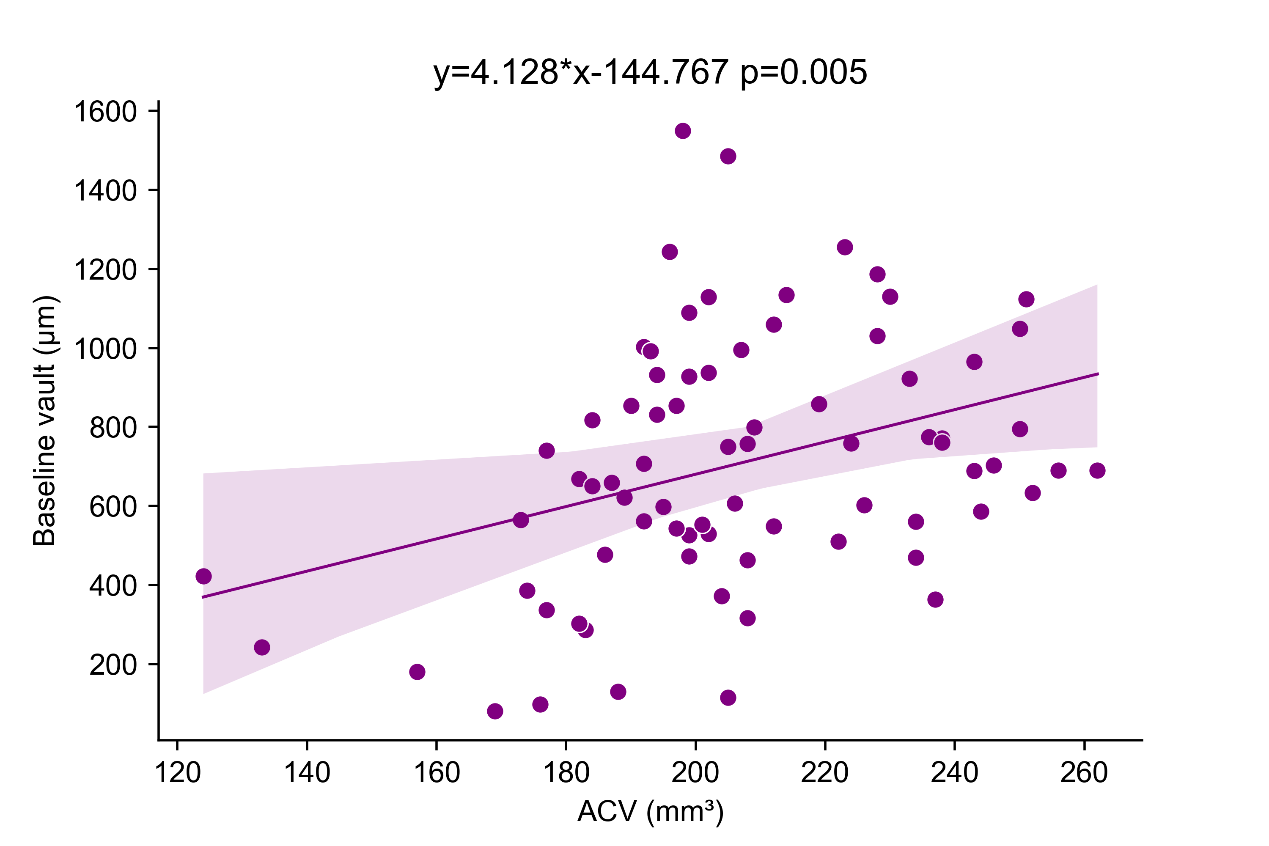


**Figure B.** Scatter plot demonstrating correlations between baseline vault and ACV. A 10 mm³ increase in ACV was associated with a 4.13 μm crease in baseline vault. ACV：anterior chamber volume.


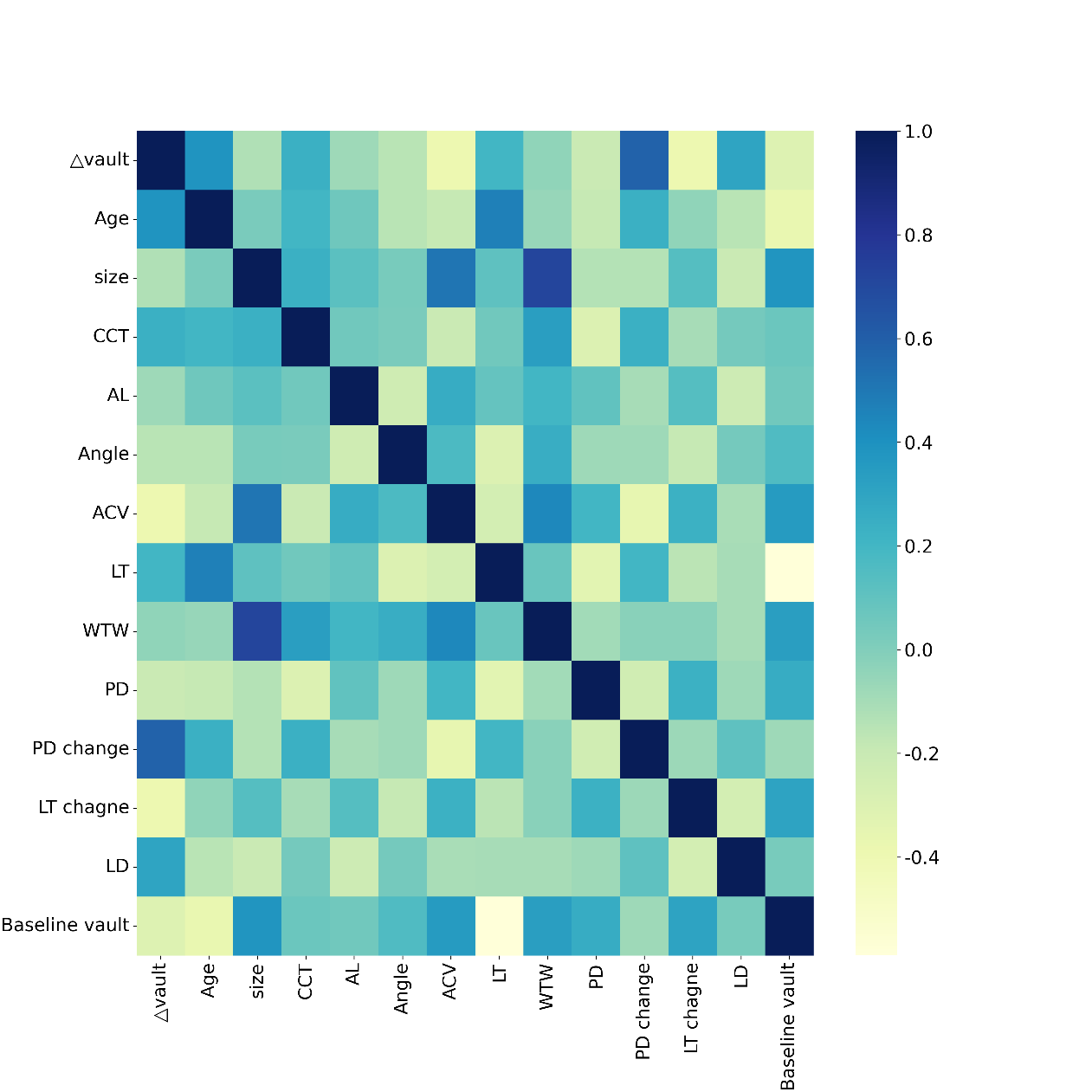


**Figure C.** Heatmap demonstrating correlations among ocular parameters. ICL = implantable collamer lens; CCT = central corneal thickness; AL = axial length; ACV = anterior chamber volume; WTW = white-to-white; LT = lens thickness; PD = pupil diameter. LD: lens displacement.
