# Supplementary figures and images for: Effect of lighting conditions on implantable collamer lens vault: Influence of anterior chamber and lens parameters
Source: Heliyon. 2024 Sep 13;10(18):e37895. doi: 10.1016/j.heliyon.2024.e37895 (PMC11420470; doi:10.1016/j.heliyon.2024.e37895)

## Slide 1
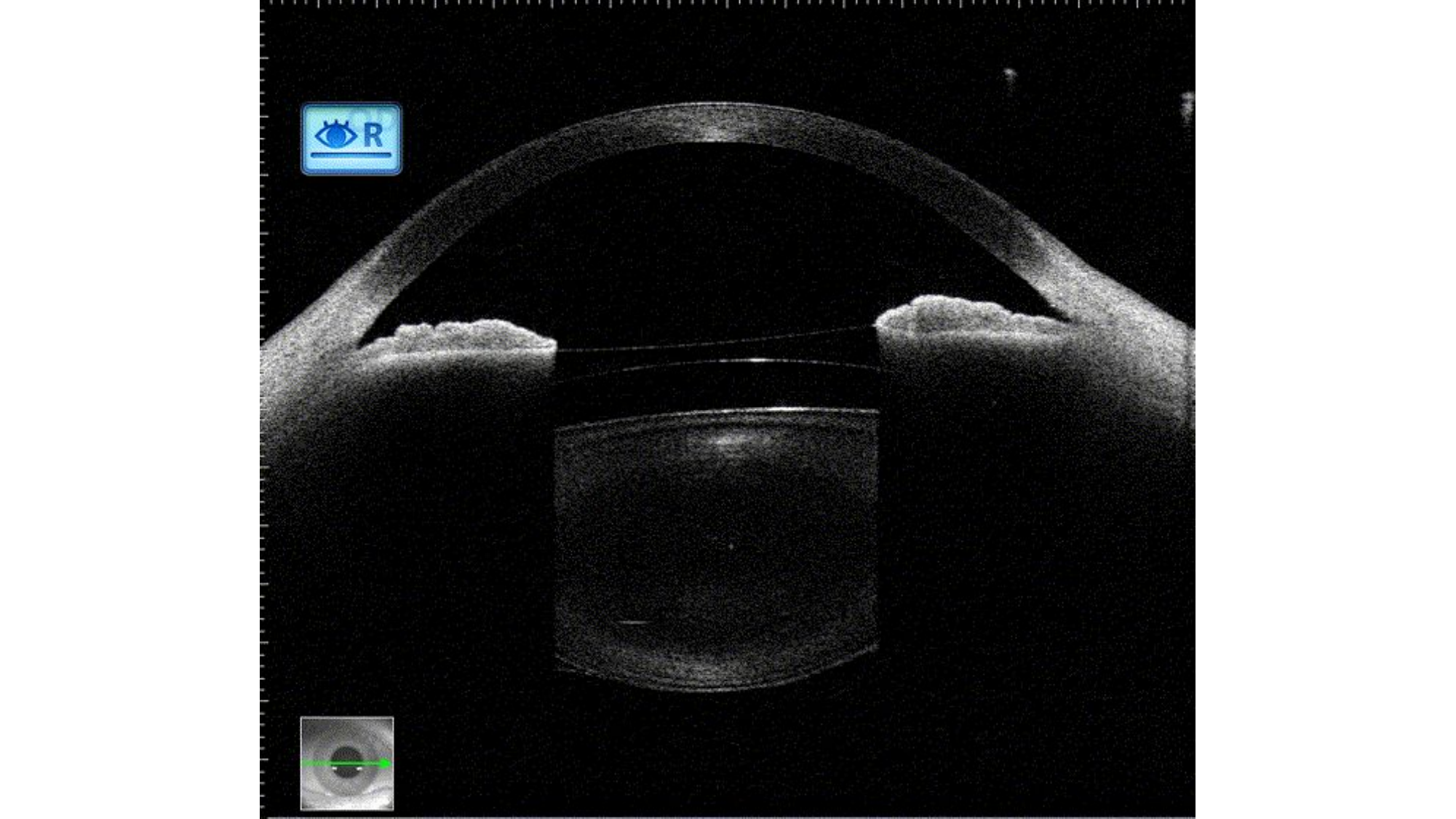

Supplement: Multimedia component 2 [file mmc2.pptx]
